# Isolation of *Chryseobacterium siluri* sp. nov., from liver of diseased catfish (*Silurus asotus*)

**DOI:** 10.1016/j.heliyon.2020.e03454

**Published:** 2020-02-22

**Authors:** Woo Taek Oh, Jin Woo Jun, Sib Sankar Giri, Saekil Yun, Hyoun Joong Kim, Sang Guen Kim, Sang Wha Kim, Se Jin Han, Jun Kwon, Se Chang Park

**Affiliations:** a Laboratory of Aquatic Biomedicine, College of Veterinary Medicine and Research Institute for Veterinary Science, Seoul National University, Seoul 08826, Republic of Korea; b Department of Aquaculture, Korea National College of Agriculture and Fisheries, Jeonju 54874, Republic of Korea

**Keywords:** Microbiology, Bacteria, Microbial genomics, Microorganism, Microbial biotechnology, Epidemiology, *Chryseobacterium*, Catfish, Phylogeny, South Korea

## Abstract

Yellow-pigmented, circular bacteria (strain SNU WT7) were isolated from the liver of moribund eastern catfish (*Silurus asotus*). Our study focused on the taxonomic description of SNU WT7 using phylogenetic, phenotypic, and chemotaxonomic analyses. The 16S rRNA gene sequence of the SNU WT7 strain was highly similar to that of *Chryseobacterium haifense* H38^T^ (97.29% similarity), followed by *Chryseobacterium hominis* P2K6^T^ (97.22% similarity), while other species exhibited similarity values of less than 97.0%. The genome of strain SNU WT7 displayed average nucleotide identity and genome-to-genome distance values of 72.35% and 22.0%, respectively, which clearly indicated that the novel species was distant from the other *Chryseobacterium* species, with its closest relative being *C. haifense* H38^T^. Furthermore, the phenotypic characteristics, including acid production from glucose, D-fructose, lactose, and maltose, of strain SNU WT 7 differed from those of *C. haifense* H38^T^. The major polar lipid of the strain was phosphatidylethanolamine, and several unidentified aminolipids and lipids were also present. Similar to other *Chryseobacterium* species, the quinone system was composed mainly of MK-6. The genome of SNU WT7 is 2,690,367 bp with a G + C content of 43.6%. Taken together, our data indicate that the isolate SNU WT7 represents a novel species of the genus *Chryseobacterium*. Thus, we present the name *Chryseobacterium siluri* sp. nov. for the novel type strain SNU WT7^T^ (KCTC 72626, JCM 33707).

## 1 Introduction

The genus *Chryseobacterium*, which belongs to class Flavobacteriia and family *Flavobacteriaceae*, was first described by Vandamme et al. in 1994 [1]. *Chryseobacterium* spp. are gram-negative, aerobic, and oxidase- and catalase-positive bacteria. The genus comprises more than 100 species (http://www.bacterio.net/chryseobacterium.html), and is found in various environments such as river water, soil, sea sediments, and food [2, 3, 4]. *Chryseobacterium* spp. are detected in both healthy as well as diseased individuals, and despite their presence in diverse environments under normal conditions, they are considered to be pathogenic and are associated with numerous infections in both humans [5] and animals [6]. *Chryseobacterium indolgenes*, which causes rare but serious infections in immune-compromised human patients, is one of the better-known pathogenic species of this genus [7]. Furthermore, the bacterium is inherently resistant to a wide spectrum of antibiotics, including beta-lactams, aminoglycosides, and linezolid [8, 9].

*Chryseobacterium* spp. are also associated with numerous infections in aquatic animals, especially fish, with various species reported to be pathogenic in different kinds of fish [6, 10]. In 1959, *C. balustinum* was the first pathogenic species to be reported for its potential role in inducing septicemia in freshwater dace (*Leucisus leucisus*) [11]. Subsequently, the pathogenicity of this bacterium was confirmed in juvenile turbot (*Scophthalmus maximus*) and rainbow trout (*Onohynchus mykiss*), as was that of *C. scophtalmum, C. viscerum*, and *C. oncorhynchi* in rainbow trout (*Onohynchus mykiss*) and *C. aahli* in lake trout (*Salvelinus namycush*) [12, 13, 14, 15]. As it exhibits resistance to multiple antibiotics, *Chryseobacterium* is considered to be a serious bacterial pathogen that threatens aquaculture [10]. In this context, we isolated a bacterial strain belonging to the genus *Chryseobacterium* from a catfish (*Silurus asotus*) farm in Korea. Our study suggested the strain to be one of the novel species among *Chryseobacterium* group based on the analysis result of its 16S rRNA gene sequence, phylogenetic study, whole genome sequence, and phenotypic characteristics. Comprehensively, the analysis results meet the criteria for classification in novel species of bacteria and our study present *C. siluri* as a novel bacterial species isolated from Korean catfish (*Silurus asotus*).

## 2 Material and methods

### 2.1 Isolation and ecology

Herein, we report a novel *Chryseobacterium* strain, SNU WT7, isolated from the liver of the diseased catfish, *Silurus asotus*. Moribund catfish were collected from one of the catfish farm located in Korea and their internal organs including kidney, liver and spleen was extracted for the isolation of the pathogen. These procedure followed ethical rules for handling fishes but there are no detailed ethic rules and regulations for market sale deceased fish in Korea. Post mortem examination can be done by person who has D.V.M degree in our department legally. For ethical statement, we've followed ethical guidelines from institutional animal care and use committee (IACUC) of Seoul National University. Also we've followed ARRIVE guidelines and used 3Rs on examination procedure on animal work. Here is the link for Institutional Animal Care and Use committee of Seoul National University, you can see more detailed regulations here; https://iacuc.snu.ac.kr/index.htm?#

Following these procedures, each organ was streaked for bacterial cultivation and the following strain was cultured on tryptic soy agar (TSA; BD Difco, Sparks, MA, USA) plates at 25 °C for 24 h until round, oval-shaped, and yellow-pigmented colonies were observed. The bacteria were sub-cultured on the same agar for isolation of pure culture, and then stored in tryptic soy broth (TSB; BD, Difco, Sparks, MA, USA) supplemented with 30% glycerol at −80 °C. For further analysis of the strain, pure cultured bacterial isolates were maintained on TSA for biochemical and chemotaxonomic characterization.

### 2.2 16S rRNA phylogeny for identification

**Figure 1:**
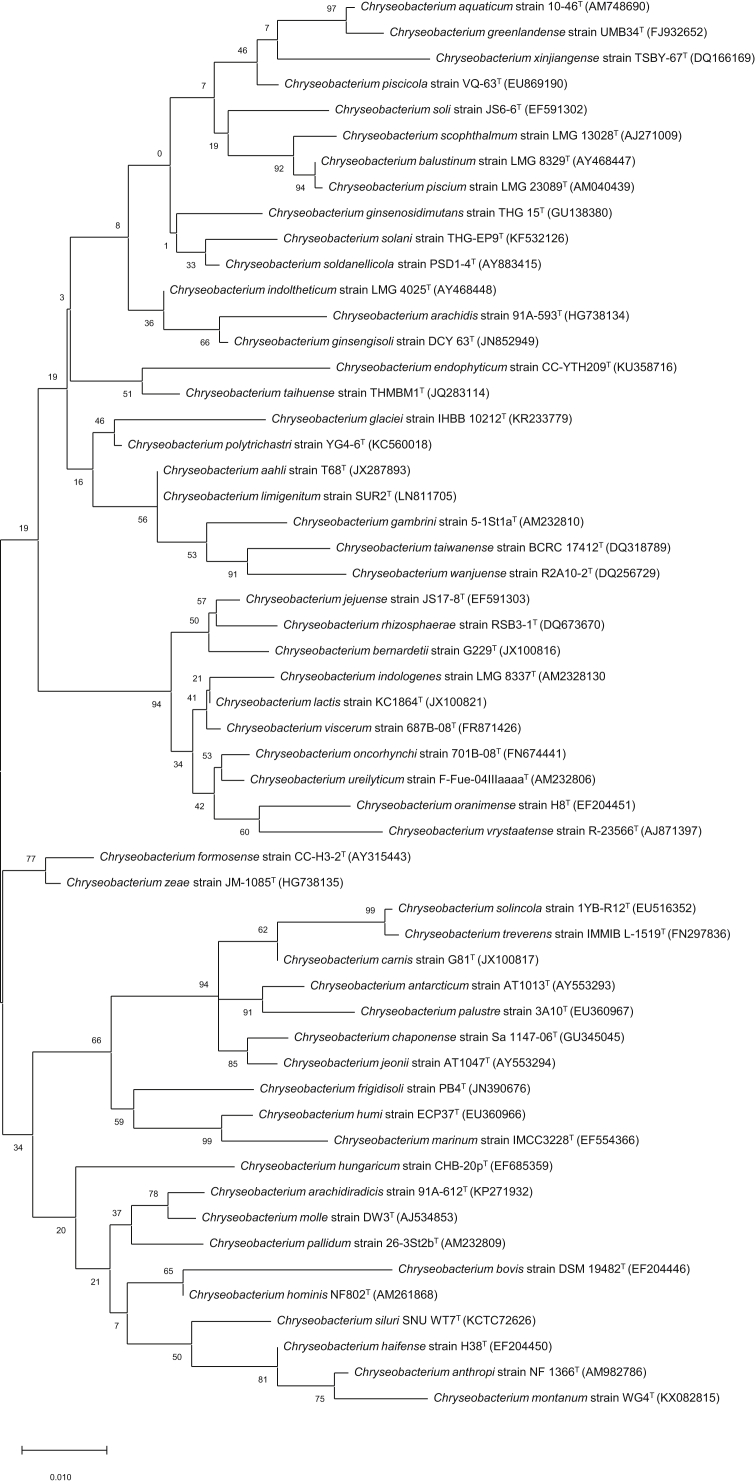
Phylogenetic tree, constructed using maximum-likelihood method via MEGA 7.0 software with bootstrap replicates of 1000. A total of 56 partial 16S rRNA sequences were used (1413 bp) (GenBank accession number: MN519493). Bar indicates 0.01 changes per nucleotide position.

For bacterial identification purposes, 16S rRNA gene sequencing was performed using PCR using the specific primers 27F and 1492R, following the protocol described by Frank et al. [16]. PCR products were forwarded to the genomic division of Macrogen Inc. (Daejeon, Korea), where nucleotide sequencing reactions were carried out using an ABI PRISM 3730XL DNA analyzer and BigDye® Terminator v3.1 Cycle Sequencing Kit (Applied Biosystems, USA). The obtained results were compared with those of other 16S rRNA genes using EzTaxon server (https://www.ezbiocloud.net/identify) which compares 16S rRNA genes of bacterial species using blastN tool. For phylogenetic analysis, 55 *Chryseobacterium* 16S rRNA gene sequences were collected and imported into BioEdit software [17] for alignment. MEGA 7.0 software was then used to construct a phylogenetic tree from the aligned sequences [18]. Analysis was performed using the maximum-likelihood method with a bootstrap of 1000 replicates (Figure 1).

### 2.3 Complete genome sequence and genome comparison

The complete genome of strain SNU WT7 (GenBank accession number: CP044507) was sequenced by Macrogen Inc. Korea, via a hybrid approach using the PacBio RS II system (Pacific Biosciences, USA) and HiSeq 2000 platform (Illumina, USA). Analysis of the genome features of strain SNU WT7 was performed by comparing the average nucleotide identity (ANI) values between closely related bacterial strains. ANI values were obtained via an ANI calculator that used the OrthoANIu algorithm tool (https://www.ezbiocloud.net/tools/ani). In addition, the intergenomic distances between SNU WT7 and species closely related to it were calculated using GGDC v2.1 (DSMZ, http://ggdc.dsmz.de/ggdc.php#) [19].

### 2.4 Phenotypic characterization

Cultural and morphological characteristics of the strain were recorded based on cultures grown on TSA at 25 °C for 24 h. Gram staining was performed using a Gram staining kit (BioMerieux, Durham, NC, USA) according to the manufacturer's protocol. For motility tests, the strain was cultured on TSB at 25 °C and observed under a light microscope. The oxidase reaction was assessed with an oxidase reagent (BioMerieux, Durham, NC, USA) according to the manufacturer's protocol. Catalase activity was observed by gas formation following a hydrogen peroxide drop. Bacterial growth was observed under conditions ranging from 4 °C to 42 °C on TSA and MacConkey agar. For NaCl tolerance, bacterial growth was examined on TSB containing a concentration of 0–10% NaCl. To estimate the biochemical characteristics and physiological patterns of the strain, SNU WT7 was cultured on TSA at 25 °C for 24 h and assessed using API 20E, API 20NE, API ZYM, and API CH kits (BioMerieux, Durham, NC, USA).

Fatty acid methyl ester analysis was performed by the Korean Culture Center of Microorganisms (KCCM). SNU WT7 was grown on TSA at 25 °C for 24 h, extracted according to the Sherlock Microbial Identification System (MIDI) protocol and analyzed using a Hewlett Packard HP 6890 gas chromatograph. High pressure liquid chromatography (HPLC) analysis was used to determine the composition of polar lipids and quinones in SNU WT7. The strain was cultured on TSB at 25 °C for 48 h prior to HPLC analysis.

The GenBank accession number of the complete genome sequence of strain SNU WT7 is CP044507 and its 16S rRNA has been registered under the GenBank accession number MN519493.

## 3 Results and discussion

The comparison result of 16S rRNA gene of strain SNU WT 7 with its close relatives by using EzBio Cloud server database implicated distinct result. Strain SNU WT 7 exhibited the highest similarity to *C. haifense* H38^T^ (97.29% sequence similarity), followed by *C. hominis* DSM 19326^T^ (97.22%), *C. pallidum* DSM 18015^T^ (96.73%), and *C. molle* DSM 18016^T^ (96.59%). In addition, the resulting phylogenetic tree constructed using MEGA software was similar to those on the EzTaxon server, wherein strain SNU WT 7 was closely related to *C. haifense* H38^T^ and *C. montanum* WG4^T^ (Figure 1). However, strain SNU WT7 clearly formed a clade that was separate from the original group, thereby representing a novel species within the *Chryseobacterium* group.

The genome of strain SNU WT7 consisted of two circular contigs, representing the genome of the bacterial strain (2,690,367 bp) and the sequence of the plasmid (67,029 bp), respectively. The sequencing depth of the genome was calculated as 81.27. The estimated ANI value between strain SNU WT7 and *C. haifense* DSM 19056^T^ was 72.35, while those with *C. hominis* DSM 19326^T^, *C. arachidiradicis* DSM 27620^T^, *C. bovis* DSM 19482^T^, *C. koreense* CCUG 49689^T^, and *C. molle* DSM 18016^T^ were 70.25, 70.14, 71.10, 71.09, and 70.22, respectively (Table 1). The estimated values were clearly below the species cut off value of 95% [20], further indicating that strain SNU WT7 was a novel species of the *Chryseobacterium* genus.

**Table 1:** Comparison of average nucleotide identity (ANI) values between *C. siluri* KCTC 72626^T^ and closely related *Chryseobacterium* species.

	*C. siluri* KCTC 72626^T^	*C. haifense* DSM 19056^T^	*C. hominis* DSM 19326^T^	*C. arachidiradicis* DSM 27620^T^	*C. bovis* DSM 19482^T^	*C. koreense* CCUG 49689^T^	*C. molle DSM* 18016^T^
*C. siluri* KCTC 72626^T^	-	72.35	70.25	70.14	71.10	71.09	70.22
*C. haifense* DSM 19056^T^	72.35	-	72.72	72.70	74.85	75.97	73.33
*C. hominis* DSM 19326	72.35	72.72	-	82.48	78.65	71.91	80.80
*C. arachidiradicis* DSM 27620	70.14	72.70	82.48	-	78.42	71.89	81.11
*C. bovis* DSM 19482^T^	71.10	74.85	78.65	78.42	-	71.84	78.00
*C. koreense* CCUG 49689^T^	71.09	75.97	71.91	71.89	71.84	-	72.43
*C. molle DSM* 18016^T^	70.22	73.33	80.80	81.11	78.00	72.43	-

The genomic distances were 22% with *C. haifense* DSM 19056^T^, and 20.5%, 19%, 23.8%, 18.9%, and 18.9% with *C. hominis* DSM 19326^T^, *C. arachidiradicis* DSM 27620^T^, *C. bovis* DSM 19482^T^, *C. koreense* CCUG 49689^T^, and *C. molle* DSM 18016^T^, respectively (Table 2). All distances were clearly below the cut off value of 70% for bacterial species and 79% for subspecies [21]. Moreover, the difference in G + C content between the strains was 6.7, 7.79, 7.37, 5.33, 3.43, and 6.1 in case of *C. haifense* DSM 19056^T^, *C. hominis* DSM 19326^T^, *C. arachidiradicis* DSM 27620^T^, *C. bovis* DSM 19482^T^, *C. koreense* CCUG 49689^T^, and *C. molle* DSM 18016^T^, respectively (Table 2). Together, these results suggest that SNU WT7 can be considered as a novel species of the genus *Chryseobacterium*.

**Table 2:** GGDC result analyzed via GGDC V 2.1 (http://GGdc.dsmz.de) between *C. siluri* KCTC 72626^T^ and closely related *Chryseobacterium* species.

Query genome	Reference genome	DDH	Model C.I. (Formula 2)	Distance	G + C difference
*C. siluri* KCTC 72626^T^	C.haifense_DSM_19056^T^	22	[19.7–24.4%]	0.1993	6.7
*C._siluri* KCTC 72626^T^	*C. hominis*_DSM_19326^T^	20.5	[18.3–22.9%]	0.2141	7.79
*C._siluri* KCTC 72626^T^	*C. arachidiradicis*_strain_DSM_27620^T^	19	[16.8–21.4%]	0.2311	7.37
*C._siluri* KCTC 72626^T^	*C. bovis*_DSM_19482^T^	23.8	[21.5–26.3%]	0.1837	5.33
*C._siluri* KCTC 72626^T^	*C. koreense*_CCUG_49689^T^	18.9	[16.7–21.3%]	0.233	3.43
*C._siluri* KCTC 72626^T^	*C. molle*_strain_DSM_18016^T^	18.9	[16.8–21.3%]	0.2323	6.1

In physiology and chemotaxonomic analysis, SNU WT7 was found to be gram-negative, non-motile, oxidase- and catalase-positive, rod-shaped, and 1 μm in width and 2–3 μm in length. Growth was observed on both TSA and MacConkey agar under conditions ranging from 4 °C to 37 °C and NaCl tolerance was measured to be 3.0%.

Phenotypic characteristics of the strain were ascertained by monitoring acid production from glucose, D-fructose, lactose, and maltose. Following result of acid production characteristics can be differentiated when compared with those of close relatives such as *C. haifense* DSM 19056^T^, *C. anthropi* NF 1366^T^, and *C. hominis* CCUG 13649^T^. Other characteristics showed similar patterns to those of other members of the group; detailed information is presented in Table 3 [21, 22, 23, 24, 25].

**Table 3:** Comparison between phenotypical characteristics of strain SNU WT7 and its close relatives. The results were confirmed using API 20E, API 20NE, API ZYM, and API 50CH kits.

Growth on/at	*C. siluri* KCTC 72626^T^	*C. haifense* DSM 19056^T^	*C. anthropi* NF 1366^T^	*C. balustinum* ATCC 33487^T^	*C. hominis* CCUG 13649^T^
3% NaCl	+	-	+	+	-
MacConkey agar	+	-		+	-
5 °C	+	+	-	-	-
37 °C	+	+	+	+	+
42 °C	-	-	-	-	-
Nitrate reduction	-	-	-	+	+/-
Urease activity	-	-	-	-	-
β-Galactosidase activity	+	+	+	-	-
H_2_S production	-	-	-	-	-
Indole production	+	+	+	+	+
Acid production from:					
Glucose	-	+	-	+	+
L-Arabinose	-	-	-	NA	-
D-Fructose	-	+	-	+	NA
Lactose	-	+	-	-	-
Maltose	-	+	+	-	+
D-Mannitol	-	-	-	-	-
Trehalose	-	-	-	-	-
D-Xylose	-	-	-	-	-
DNA G + C percent (mol%)	43.6%	37.8%	39.0%	33.1%	36.5%

NA: Not available.

Fatty acid analysis result of SNU WT7 indicated that the most abundant fatty acid was iso-C_15:0_ (38.5%), followed by Anteiso-C_15:0_ (13.6%), and Iso-C_17:0_ 3-OH (12.9%). These results were similar to that of other *Chryseobacterium* species (Table 4). However, unknown fatty acids such as 13.565 and 16.582 were not detected in SNU WT7 (Table 4) [22, 23, 24, 25].

**Table 4:** Fatty acid methyl ester analysis results of strain SNU WT7 and its close relatives.

Fatty acid (%)	*C. siluri* KCTC 72626^T^	*C. montanum* WG4^T^	*C. anthropi* NF 1366^T^	*C. haifense* DSM 19056^T^	*C. koreense* KCTC 12107^T^
C_15:0_	TR	TR	TR	TR	1.3
C_16:0_	1.2	TR	TR	TR	1.1
Iso-C_13:0_	1.3	TR	1.6	1.4	1.9
Iso-C_14:0_	TR	ND	TR	ND	2.1
Iso-C_15:0_	38.5	32.4	31.9	28.9	39.2
Anteiso-C_15:0_	13.6	21.8	21.8	25.7	22.2
Iso-C_16:0_	2.7	1.3	2.5	TR	2.9
Iso-C_17:0_	TR	2.1	1.2	1.0	TR
Iso-C_17:1_*ω9c*	ND	2.2	4.6	6.9	1.7
Iso-C_15:0_ 3-OH	1.4	1.9	2.3	2.1	2.0
C_15:0_ 2-OH	TR	1.4	1.6	1.6	1.2
Iso-C_16:0_ 3-OH	1.1	2.0	1.2	TR	2.7
Iso-C_17:0_ 3-OH	12.9	17.2	16.4	14.2	10.8
C_17:0_ 2-OH	2.2	5.9	4.2	6.2	2.1
Unknown 13.565∗	ND	5.4	4.6	3.1	1.8
Unknown 16.582∗	ND	1.2	1.1	TR	TR
Summed feature 3	2.4	1.4	2.5	2.3	2.0
Summed feature 4	TR	ND	1.3	TR	ND

∗ Unknown fatty acid, number indicates equivalent chain-length. TR, Traces (<1.0 %); ND, not detected. Summed feature 3 contained C_16:1_*ω7c* and/or iso-C_15:0_ 2-OH. Summed feature 4 contained anteiso-C_17:1_ B and/or iso C_17:1_.

The polar lipid analysis result indicated that SNU WT7 contained phosphatidylethanolamine, AL 1–3 (indicating unidentified aminolipids) and L (indicating unidentified lipids) (Supplementary Figure 1). The pattern of polar lipids distribution and quinones in SNU WT7 was similar to that of other *Chryseobacterium* species [22, 23, 24, 25]. Moreover, the predominant isoprenoid quinone was estimated as MK-6.

In conclusion, the results of the phylogenetic, genomic, and phenotypic characterization demonstrated that the strain SNU WT7 may be considered as a novel species belonging to the *Chryseobacterium* genus. Considering the source of the isolate and the host of the bacterium, *Chryseobacterium siluri* sp. nov. is proposed as the generic name for this novel species.

### 3.1 Protologue

*Chryseobacterium siluri* (si.lu'ri. N.L. gen. n. *siluri* of *Silurus*, named after the catfish, *Silurus asotus*, from which the type strain was isolated) [26].

This specimen was a gram-negative, rod shaped non-motile bacterium with a width of 1 μm and a length of 2–3 μm. It grew well under aerobic conditions and was oxidase and catalase-positive. The growth temperature ranged from 4 °C to 37 °C, with optimal growth between 15 °C and 27 °C. Growth can be observed on TSA, MacConkey, and Brain Heart Infusion agar following incubation at 25 °C for 48 h. NaCl tolerance was measured as 3.0% and no growth was observed above this concentration. The shape of the bacterial colony, which was similar to that of other *Chryseobacterium* species, was circular with a diameter of 2–3 mm and the colony exhibited yellow pigmentation (Supplementary Figure 2). Phenotypic characteristics of the strain may be distinguished from other *Chryseobacterium* species through acid production from glucose, D-fructose, lactose, and maltose. Other biochemical details showed patterns similar to those of other species, such as positive reactions for indole production, Esculin hydrolysis, gelatin hydrolysis, and assimilation of glucose and maltose. In addition, negative reactions were observed for reduction of nitrates to nitrites, glucose acidification, arginine dihydrolase, urease, β-galactosidase, and assimilation of arabinose, mannose, mannitol, N-acetyl-glucosamine, gluconate, caprate, adipate, malate, citrate, and phenyl-acetate in the API 20NE test. The API ZYM test indicated that the strain was positive for alkaline phosphatase, esterase, lipase, and acid phosphatase, as well as leucine, valine, and cystine arylamidase. API ZYM also indicated negative reactions for lipase, trypsin, α-chymotrypsin, α-galactosidase, β-galactosidase, β-glucuronidase, α-glucosidase, β-glucosidase, N-acetyl-β-glucosaminidase, α-mannosidase, and α-fucosidase. Iso-C_15:0,_ anteiso-C_15:0,_ and iso-C_17:0_ 3-OH were abundant in the fatty acid profile, while Iso-C_17:1_*ω9c* was not detected. This was different to the fatty acid profiles of other close relatives. The major quinone was MK-6 and phosphatidylethanolamine, AL 1–3 (unidentified aminolipids) and L (unidentified lipid) were also identified in polar lipid analysis.

The type strain is KCTC 72626^T^ (JCM 33707^T^), isolated from the liver of catfish. The DNA G + C content of the genome of this type strain was 43.6 mol%.

## Declarations

### Author contribution statement

Woo Taek Oh: Conceived and designed the experiments; Performed the experiments; Wrote the paper.

Jin Woo Jun, Se Chang Park: Conceived and designed the experiments; Wrote the paper.

Sib Sankar Giri, Saekil Yun: Performed the experiments.

Hyoun Joong Kim, Sang Guen Kim, Sang Wha Kim: Analyzed and interpreted the data.

Se Jin Han, Jun Kwona: Contributed reagents, materials, analysis tools or data.

### Funding statement

This work was supported by the Cooperative Research Programme for Agriculture Science and Technology Development (Supportive Managing Project of Centre for Companion Animals Research) by Rural Development Administration (PJ013985032018).

### Competing interest statement

The authors declare no conflict of interest.

### Additional information

Data associated with this study has been deposited at GenBank under the accession numbers CP044507 and MN519493.

Supplementary content related to this article has been published online at https://doi.org/10.1016/j.heliyon.2020.e03454.

## Appendix A Supplementary data

Supplementary figure 1 Result of *C. siluri* KCTC 72626^T^ polar lipid analysis. PE: Phosphatidylethanolamine, AL1-3: Unidentified aminolipids, L: Unidentified lipid Supplementary figure 1

Supplementary figure 2 The colonies of *C. siluri* grown on 25 °C TSA plate for 36h depicting yellow pigmentation with oval shape. Supplementary figure 2
