# Supplementary figures and images for: Isolation of Chryseobacterium siluri sp. nov., from liver of diseased catfish (Silurus asotus)
Source: Heliyon. 2020 Feb 22;6(2):e03454. doi: 10.1016/j.heliyon.2020.e03454 (PMC7036476; doi:10.1016/j.heliyon.2020.e03454)

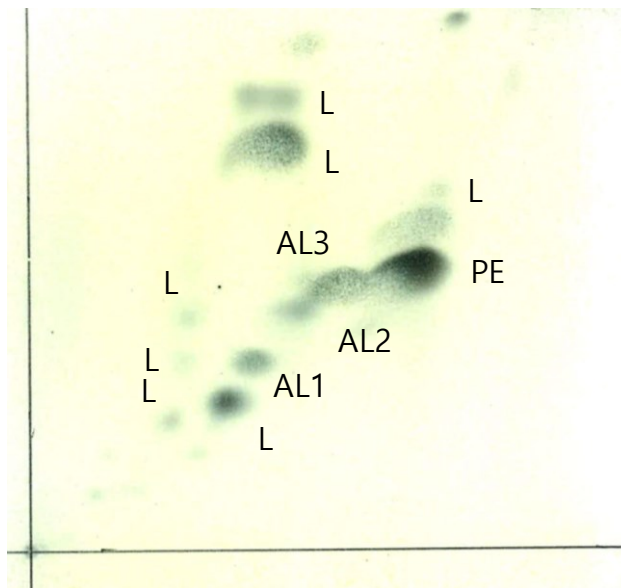

Supplement: Supplementary figure 1 — Result of C. siluri KCTC 72626T polar lipid analysis. PE: Phosphatidylethanolamine, AL1-3: Unidentified aminolipids, L: Unidentified lipid [file mmc1.pdf]

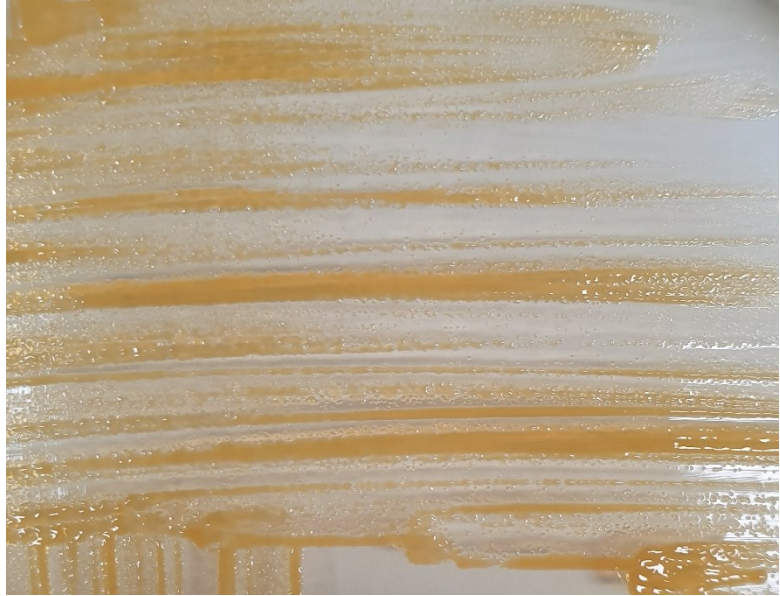

Supplement: Supplementary figure 2 — The colonies of C. siluri grown on 25 °C TSA plate for 36h depicting yellow pigmentation with oval shape. [file mmc2.pdf]
